# Geo-Mapping of Intestinal Parasitic Infection in a Southern Community in Nigeria

**DOI:** 10.4314/ejhs.v34i3.5

**Published:** 2024-05

**Authors:** J Gbonhinbor, AE Abah, GDB Awi-Waadu

**Affiliations:** 1 Department of Science Laboratory Technology, Federal Polytechnic, Ekowe, Bayelsa State, Nigeria; 2 Department of Animal and Environmental Biology, University of Port Harcourt, Rivers State, Nigeria

**Keywords:** Geo-mapping, Intestinal Parasites, Prevalence, GIS, Nigeria

## Abstract

**Background:**

Intestinal parasitic infection constitutes a global health burden; it has a high prevalence among children in Nigeria. The quest for control is still ongoing. Geographical Information Systems have contributed significantly to solving sundry real-world tasks, from agriculture to emergency planning and control Therefore, this study was aimed at geo-mapping of intestinal parasites in a Southern community in Nigeria to identify the infection risk areas.

**Methods:**

A cross-sectional survey and clustered random sampling method were used. Samples were analyzed by direct wet mount and formal ether concentration methods. Geostatistical analyses were done to determine the spatial distribution of these parasites.

**Results:**

The overall prevalence of intestinal parasite in the community was 23.95% and parasites identified were: Ascaris lumbricoides 45(7.23%), Entamoeba histolytica 31(4.98%), Strongyloides stercoralis 13(2.09%), Gardia lambla 12(1.93%), Hookworm 11(1.77%), Trichuris trichiura 10(1.61%), Schistosoma mansoni 9(1.45%) and Diphyllobothrium latum 4(0.64%). The distribution and intensity of the parasites showed that Bolu-Orua, Tungbo, and Ogalawa communities had higher intestinal parasitic infection rates and needs urgent interventions. Part of Sagbama, Aguru, Toru-Orua to Toru-Eden had a moderate intestinal parasitic infection.

**Conclusion:**

An infection map was produced for each parasite, and visualizing the spatial distribution of intestinal parasites in these communities brings to bare health risk areas. It will help in the proper application of limited resources in the control and prevention of these parasites.

## Introduction

Intestinal Parasitic infections are among the most common infections worldwide and affect the poorest and most deprived communities. They are transmitted by eggs present in human faeces which in turn contaminate soil in areas where sanitation is poor (1).

More than 1.5 billion people, 24% of the world's population, are infected with these parasitic infections worldwide (1). Infections are widely distributed in tropical and subtropical areas, with the greatest numbers occurring in sub-Saharan Africa. However, one of the WHO 2030 global targets for this infection is to achieve and maintain the elimination of the parasites' morbidity in preschool and school-age children (2).

Grievous and widespread as these infections are, they are categorized as one of the neglected tropical diseases because not serious attention is given to them by many States unlike Malaria, HIV, and the recent COVID-19. Due to the susceptibility of children, intestinal parasitic infection is a common occurrence among them (3).

The application of Geographical Information Systems (GIS) has contributed significantly to solving sundry real-world tasks, from agriculture to emergency planning and control. It has been used in the health sector for infectious diseases and other parasitic diseases in Africa and the world over (4). For example, it was deployed for the African Programme for Onchocerciasis Control (APOC) where it was used effectively to picture priority areas for mass distribution of Invermetin and determine the number of people to be treated (4). In control and intervention, mapping helps to guide available resources to be most rationally and cost-effectively deployed (5).

Intestinal parasitic infection constitutes a global health burden, and its high prevalence among children in Nigeria has been reported because of their vulnerability (3,6,7). Yet, information on the geo-mapping of intestinal parasites in Nigeria is scanty, and such data are not readily available in Bayelsa State. Also, to achieve the WHO 2030 global target of elimination of these parasite morbidity in school children, conscious efforts must be made to geo-map these parasites for ease of control. Therefore, this study aimed to determine the spatial distribution of intestinal parasitic infection in Sagbama Local Government Area of Bayelsa State.

## Materials and Methods

**Figure 1:**
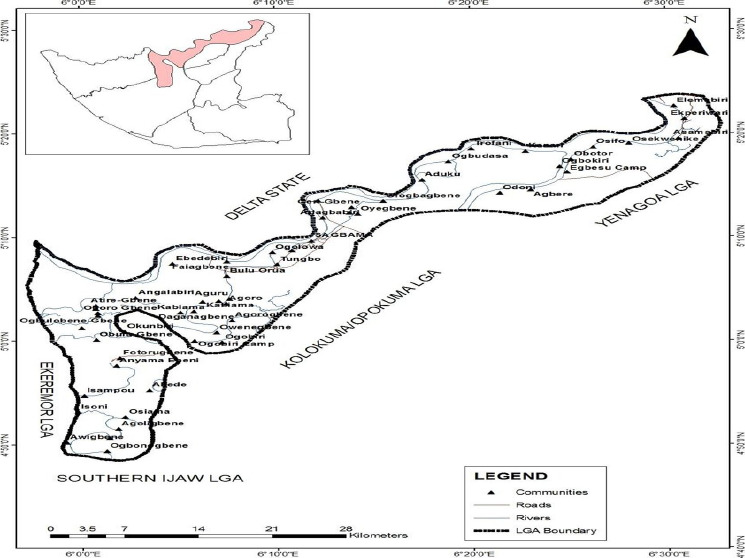
Map of the studied communities in Bayelsa State

**Study area**: Sagbama Local Government Area has coordinates 10°6/N 6°12/E. It has an area of 945km^3^ with an estimated population of 187, 146 (8). The study was carried out in nine riverine communities (Figure 1). These communities are rural in setting and residences are built in block houses, clustered homesteads of mainly mud homes, enforced with bamboo sticks. Sagbama climate and vegetation are consistent with that of a typical rainforest region in Southern Nigeria. These villages have a broad coastal plain topography with many ponds, streams, and a river. Bayelsa State experiences heavy rainfall from May to October with its peak in August. Dry seasons start from November to April. The average temperature of the area is about 25-34^0^C. Crops grown in these communities include sugarcane, banana, plantain, cassava, yam, beans, garden egg, fresh tomatoes, fresh pepper, cucumber, groundnut, okro, cocoyam, water yam, and vegetables which are planted and cultivated in large quantity basically for consumption and commercial purposes. The majority of the inhabitants in these communities make use of water from the Forcados River as a local source of drinking water, for waste disposal, faecal disposal, and other domestic activities. Inhabitants also make use of the community taps and individual bore holes as sources of drinking water. There are toilet facilities, but most inhabitants make use of the surrounding bushes, ponds and streams

**Study design**: This was a cross-sectional survey on children within the age bracket of 5-16 years at primary schools across communities in Sagbama Local Government Area, Bayelsa State, from March to June 2021. Clustered random sampling was used to sample the schools and primary school children that participated in this study.

**Sample collection**: A total of 622 stool samples were collected from primary school children using clean 50cm3 wide-mouthed, screw-capped universal specimen bottles. The samples were collected and processed on the same day. Samples that were not to be processed on the same day were preserved in the refrigerator. If the prevalence was more than 50% based on WHO recommendation, the school children would be dewormed. All samples were collected with structured questionnaires requesting some basic epidemiological information. The specimens were labelled appropriately on submission of stool samples and properly corked. On-the-spot macroscopic analysis was done using the direct technique for consistency, blood, mucus, and motile trophozoites. The samples were placed in a cooling container and then taken to the Parasitology Research Laboratory of the Department of Animal and Environmental Biology, University of Port Harcourt for examination and further analysis.

**Stool sample analysis**: The samples were analyzed by direct wet mount and formal ether concentration method as described by Cheesbrough (9). GIS Technique and method used for creating map.

The data were collected using global positioning system equipment to sample the locations of parameters retrieved across the study area. These parameters were assigned to the Geolocations in the study areas as derived and analyzed in the GIS environment using the special analytical tool of Inverse Distance Weighted (IDW) method of interpolation to show or create an idea of the spatial spread of each phenomenon measured across space (4).

**Figure 2:**
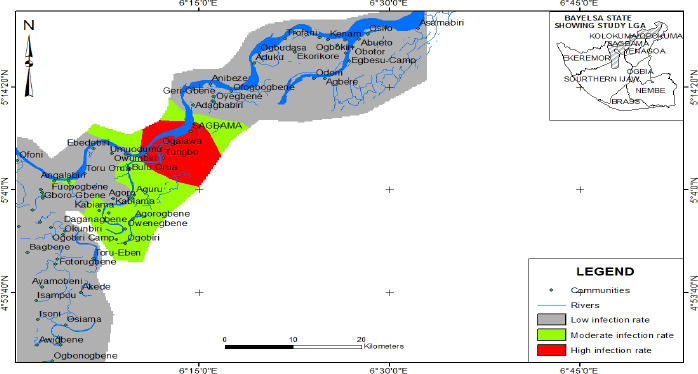
Map of Infections across sampled Communities in Sagbama LGA, Bayelsa State

**Mapping of intestinal parasitic infection in study area**: The Co-ordinates (latitude and longitude) obtained using the field Model GPS (Garmin GPS MAP76s Chart plotting receiver) were converted from degrees/minutes/seconds to decimal degrees giving the values for easting and northing. The values were displayed in Arc map/Arc info version 10.0, and this gave the location of the schools (sampling sites, Figure 1) rate of infections. Conversion of the rate of infections from the percentage value using the Geostatistical analysis wizard produced the map of infection (Figure 2). The map result showed the areas infected with different parasites.

The map was produced using Geostatistical analyst extension in ArcGIS 10.

**Consent and approval**: Ethical approval was obtained from the Department of Public Health, the State Ministry of Health and the State Primary Schools Board. Written informed consents were obtained from the Community Development Committee (CDC), and informed consents were also given by community chiefs, elders, parents and guardians before the study. The parents, teachers and participants were properly informed about the aims, objectives, benefits and protocols of the study, the need for voluntary participation and the right to stop participation at any time.

## Results

Out of the total population of primary school children, 622 submitted samples for coproparasitological examination; 335(53.85%) were males while 287(46.15%) were females. The overall prevalence was 149 (23.95%) which is the number that tested positive for intestinal parasites, and of this, 25.07% prevalence was recorded for males and 22.65% for females. Populations examined based on the communities showed that more participants were examined in Bulou-Orua (133) followed by Sagbama (128), and Angalabiri (106) with Adagbabiri (23) having the least number of participants. The result recorded showed a positive prevalence and intensity of intestinal parasites with *Ascaris lumbricoides* 45(7.23%), *Entamoeba histolytica* 31(4.98%), *Strongyloides stercoralis* 13(2.09%), *Gardia lambla* 12(1.93%), Hookworm 11(1.77%), *Trichuris trichiura* 10(1.61%), and *Diphyllobothrium latum* 4(0.64%) in this order (Table 1).

**Table 1:** Prevalence of Intestinal parasites among primary school children in primary schools across different communities in Sagbama Local Government Area, Bayelsa State, Nigeria

Communities	No. examined	No. infected	Intestinal parasites species (%)										Prevalence (%)

			*A.* *lumbricoides*	*E.* *histolytica*	Hookworm	*G.* *Lamblia*	*S.* *stercoralis*	*T.* *trichiura*	*D. latum*	*F.* *hepatica*	*S.* *mansoni*	Mixed infection	
Adagbabiri	23	11	3	3	0	0	3	1	0	0	1	3	47.83
Angalabiri	106	21	2	3	0	0	1	1	0	0	2	4	19.81
Bulou-Orua	133	20	7	3	2	5	1	1	0	0	1	6	15.04
Ebedebiri	74	14	3	3	3	0	1	2	1	1	0	2	18.92
Ofoni	33	3	2	1	1	1	0	0	0	0	0	0	9.09
Sagbama	128	48	16	12	5	6	2	3	3	0	1	6	37.50
Toru-Angiama	37	6	3	2	0	0	1	0	0	0	0	0	16.22
Toru-Orua	51	14	3	2	0	0	4	1	0	1	3	3	27.50
Trofani	37	12	6	2	2	0	0	1	0	0	1	3	32.43
**Total**	**622**	**149**	**45(7.2%)**	**31(5.0%)**	**11(1.8%)**	**12(1.9%)**	**13(2.1%)**	**10(1.6%)**	**4(0.6%)**	**2(0.3%)**	**9(1.5%)**	**28(4.5%)**	**23.95**

For species intensity, *A.lumbricoides* had the highest intensity in Sagbama at 8, followed by Angalabiri at 4 and Bolou-Orua at 3.5. This was followed *E. histolytica at* 5.5±3.536 in Sagbama, 3±0.000 in Angalabiri and 1.5±0.707 in Adagbabiri, Bolou-Orua and Ebedebiri respectively. The intensity of parasites across various communities is in the descending order of *A. lumbricoides>E. histolytica>*Hookworm*>G.Lamblia>S.stercoralis>T. trichiura>S. mansoni>D. latum>F. hepatica*. (Table 2).

**Table 2:** Distribution and identification of Intestinal parasites among primary school children across different communities in Sagbama Local Government Area, Bayelsa State, Nigeria

Communities	*Mean Intensity*								

	*A.* *Lumbricoides*	*E.* *Histolytica*	Hookworm	*G.* *Lamblia*	*S. stercoralis*	*T. trichuria*	*D. latum*	*F. hepatica*	*S. mansoni*
Adagbabiri	1.5±0.707	1.5±0.707	0	0	1.5±2.121	0.5±0.707	0	0	0.5±0.707
Angalabiri	4±2.828	**3**±0.000	**0.5**±0.707	0.000	**0.5**±0.707	**1.5**±0.707	0.000	0.000	**1**±1.414
Bulou-Orua	3.5±0.707	1.5±0.707	1±1.414	2.5±2.121	0.5±0.707	0.5±0.707	0.000	0.000	0.5±0.707
Ebedebiri	1.5±0.707	1.5±0.707	1.5±2.121	0.000	0.5±0.707	1±0.000	0.5±0.707	0.5±0.707	0.000
Ofoni	**1**±0	**0.5**±0.707	0	0	0	0	0	0	0
Sagbama	8±4.243	**5.5**±3.536	**2.5**±2.121	**3**±0.00	**1.5**±0.707	**1.5**±0.707	**1.5**±2.121	0.00	**0.5**±0.707
Toru-Angiama	1.5±2.121	1±0.000	0±0.000	0±0.000	0.5±0.707	0±0.000	0±0.000	0±0.000	0±0.000
Toru-Orua	1.5±0.707	1±0.000	0±0.000	0±0.000	2±1.414	0.5±0.707	0±0.000	0.5±0.707	1.5±2.121
Trofani	2.5±0.707	1±0.000	1.5±0.707	0±0.00	0±0.000	0.5±0.707	0.00	0.00	0.5±0.707

**Figure 3:**
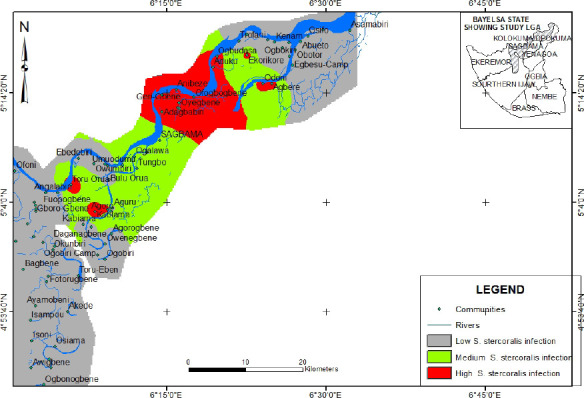
Infection map of S. stercoralis across sampled communities in Sagbama L.G.A

The map result showed the areas infected with different parasites. Part of Bulu-Orua, Tungbo and Ogolowa communities had the highest rate of infection with above 41%, followed by Part of Sagbama, Aguru, Toru Orua, Ogobiri to Toru - Eben with moderate infection while communities like Ayamobeni, Akede, Ebedebiri to the south and Trofani, Agbere and Adagbabiri to the north, all had low infection rate (Figure 2). Concerning specific intestinal parasites distribution, *Ascaris lumbricoides, E.histolytica*, hookworm, *G.lamblia, T.trichiura* and *D.latum* showed a similar pattern except *S.stercoralis* which has the highest infection rate in Adagbabirir, Anibeze, Ogebene and its environments (Figure 3).

## Discussion

The prevalence of of intestinal parasitic infection (23.95%) recorded in this study was considered to be relatively high (10). This is because this finding falls in between what was previously reported around that axis in different studies (11,12). The study area in the present study is slightly more rural than those in the previous studies and, therefore may account for the slight variation.

*Ascaris lumbricoides* at 7.23% was the most prevalent of all the parasites encountered, and this is not unusual since *Ascaris* is common in low socio-economic communities where limited access to clean water, poor personal hygiene, and sanitary facilities are widespread. Also, it has been reported that an estimated 807 million–1.2 billion people in the world are infected with *Ascaris lumbricoides* (13). More so, the studied population was the school children who are the most vulnerable and more likely to exhibit poor adherence to personal hygiene. It has been established that *Ascaris* is most common in children, especially those living in rural and impoverished communities.

*Entamoeba histolytica* 4.98% was second to *Ascaris lumbricoides* among other parasites which have been described as common among people who live in areas with poor sanitary conditions in the tropics even though the distribution is worldwide. The prevalence of 4.98% of this parasite which is the causative agent of diarrhoea disease that is associated with reduced growth, impaired cognitive function, reduced vaccine efficacy, and disruption of physical and educational development in children is important (14,15). More so, according to Troeger *et al*, (16), approximately 1.6 million deaths occur each year globally due to diarrhoea with the highest burden occurring in developing countries. Though the finding was lower than 17% reported in Niger State (17), 12.6% and 17.0% in Jos, Plateau respectively all in North Central Nigeria(18,19) and 11.2% in Lagos (20). It was, however, higher than the 4.5% reported in Calabar (21). Yet, the finding deserves attention. The difference may be due to the differences in the geographical locations, better sanitation and the level of awareness by the residents.

Multiple parasitic infection of 4.50% was recorded in the present study. Concomitant parasitic infections are common in the developing world where poverty, poor personal hygiene, poor environmental hygiene and poor healthcare service providers having an inadequate supply of drug medication and inappropriate acknowledgement of the transmission systems and life-cycle styles of those parasites have been reported. The finding is worth mentioning as multiple infection increases the risk of malnutrition, anaemia, protein-energy malnutrition and growth deficits in children.

The goal of GIS was to create a rate of infections and a prediction map of the prevalence of intestinal parasitic infection in the study area (4, 22, 23). Part of Bulu-Orua, Tungbo, and Ogolowa communities had the highest rate of infection which was above 41%, followed by Part of Sagbama, Aguru, Toru Orua, Ogobiri to Toru-Eben that had moderate infection while communities like Ayamobeni, Akede, Ebedebiri to the south and Trofani, Agbere, and Adagbabiri to the north, all had low infection rate. This provides useful information for adopting more specific and targeted actions to control the parasites as geomapping has proved to be effective in more accurate identification of risk micro-areas. The maps were created using the WHO criteria. Children from Bulu-Orua, Tungbo and Ogolowa communities had an infection rate of 41-60% and were therefore classified as communities with a high prevalence of intestinal parasites. Control efforts in these communities must be intentional, including the provision of safe drinking water, school-targeted de-worming programs and health education.

A map of specific parasites in communities was also provided. This could guide future research decisions and interventions such as waste disposal plans, sanitation plans, water supply and hygiene enlightenment.

In conclusion, the visualization of the spatial distribution of intestinal parasites in these communities brings to bare health risk areas and will help in the proper application of limited resources in the control and prevention of these parasites. The map indicates that all sampled communities are at risk of intestinal parasitic infections.
